# Supplementary material for: Structure and Methyl-lysine Binding Selectivity of the HUSH Complex Subunit MPP8
Source: J Mol Biol. Author manuscript; Available in PMC 2025 Nov 18. (PMC7618375; doi:10.1016/j.jmb.2024.168890)
Supplement: Supplementary Material [file EMS210628-supplement-Supplementary_Material.zip › 1-s2.0-S0022283624005205-mmc1.pdf]

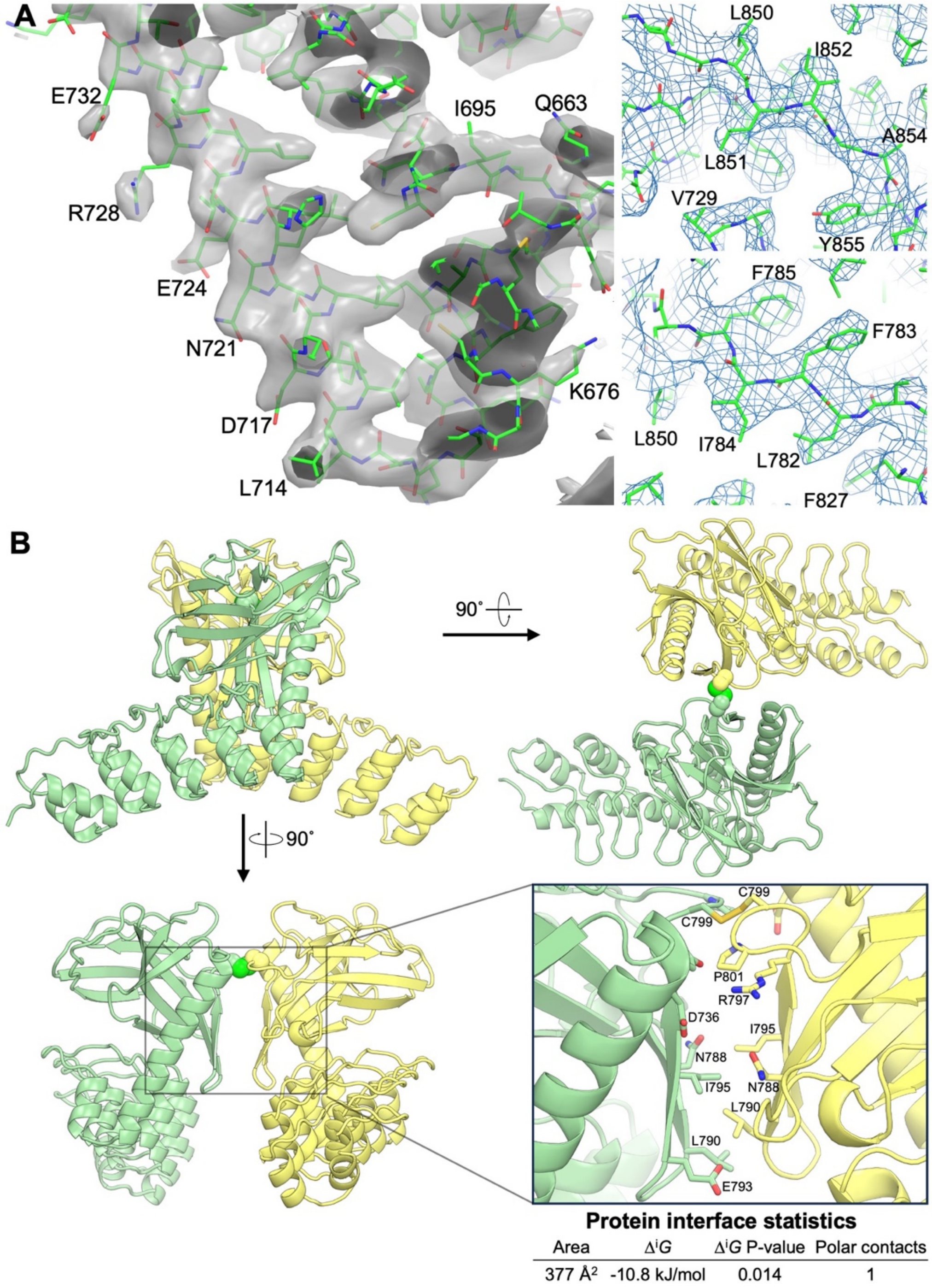

Fig. S1. Crystal structure of the MPP8 C-terminal domain (CTD). (**A**) Representative samples of the $2F_o$ – F_c electron density map of the crystal structure of MPP8 CTD. The eighth chain in the asymmetric unit (chain G) is shown. An isomesh contour level of 1.0 σ was used in PyMol Schrödinger, LLC). (**B**) One of four disulfide linked CTD homodimers in the crystallographic asymmetric unit. Inset, closeup of the dimer interface.

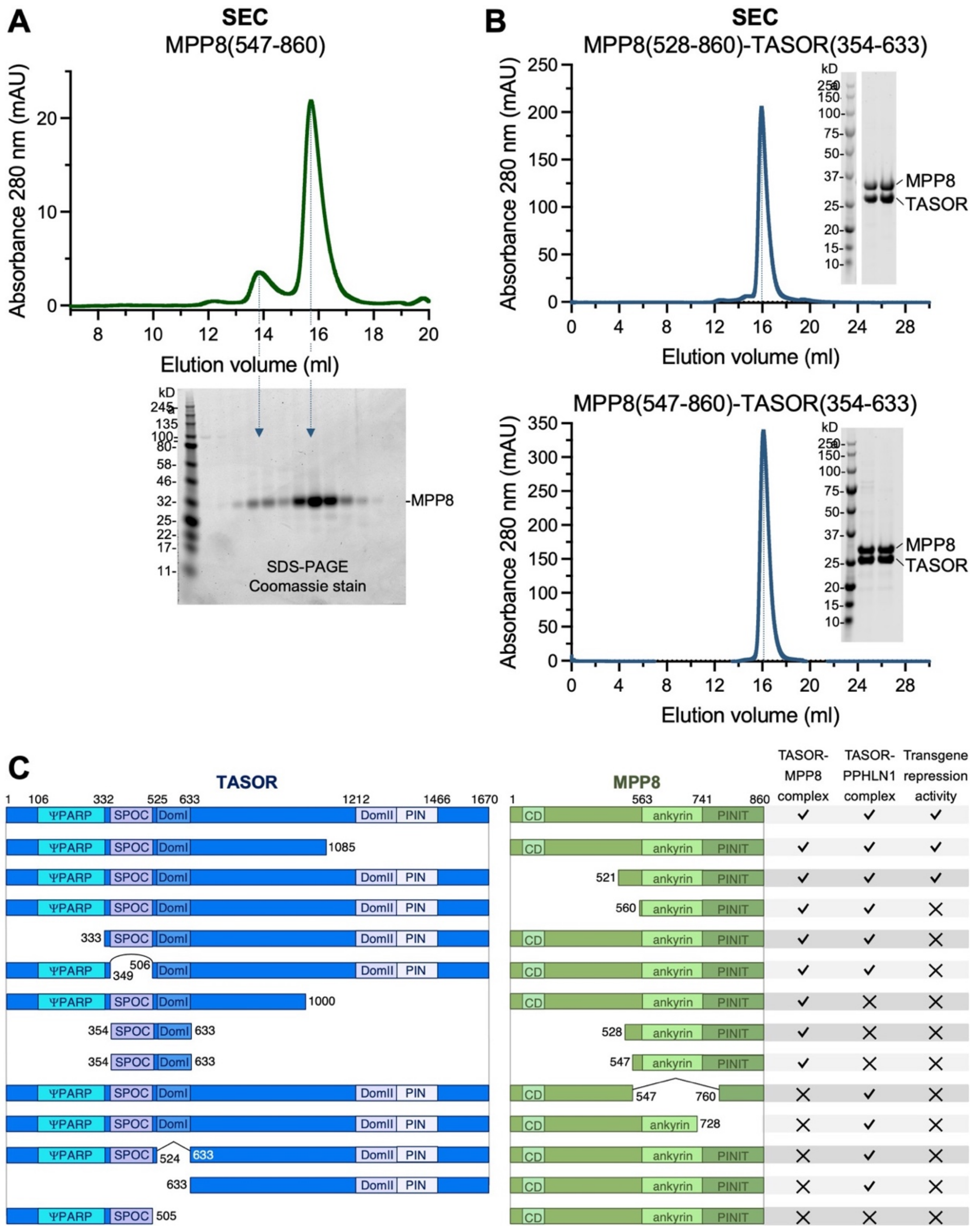

Fig. S2. Size-exclusion chromatography (SEC), coimmunoprecipitation and transgene repression activity of MPP8-TASOR complexes with different truncations. (**A**) SEC of the MPP8 C-terminal domain (CTD). The peaks at 14 ml and 16 ml elution volumes both contain pure MPP8 CTD suggesting the peaks correspond to dimer and monomer, respectively. (**B**) SEC of minimal TASOR-MPP8 complexes. (**C**) Summary of coimmunoprecipitation and transgene repression activity assay data from our previous study [14] with different MPP8 and TASOR deletion mutants. Repression activity was measured with an integrated lentivirus GFP reporter. Ticks and crosses indicate functionality in coimmunoprecipitation (complex formation) and repression assays.

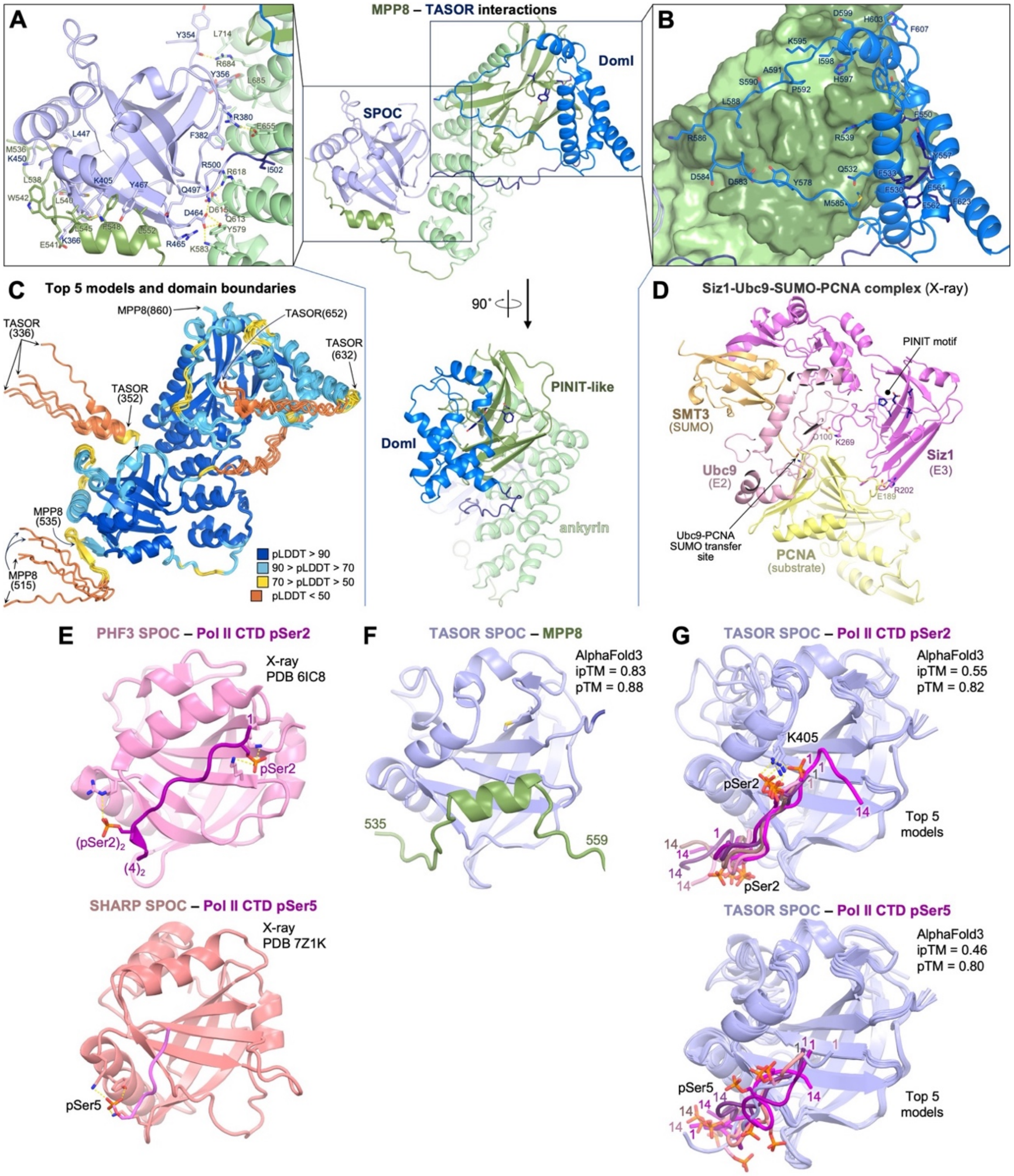

Fig. S3. Detailed properties of the AlphaFold3 model of the MPP8-TASOR complex. (**A**) Predicted interactions between MPP8 and the SPOC domain of TASOR. (**B**) Predicted interaction between MPP8 and TASOR DomI. (**C**) Superposition of the five highest scoring AlphaFold models of the MPP8-TASOR complex. Flanking sequences were included to highlight the boundaries of the folded domains (marked with arrows). (**D**) Crystal structure (PDB 5JNE) of yeast Siz1 in complex with its substrate PCNA, SUMO ortholog SMT3, and SUMO E2 ligase Ubc9 [37]. (**E**) Crystal structures of the SPOC domain from SHARP and PHF3 bound to Ser-phosphorylated RNA Pol II CTD heptapeptides [45-46]. The phosphoserine side chains are shown in stick representation. (**F**) AlphaFold model of the TASOR SPOC domain (blue) in complex with MPP8 (green). (**G**) Superposition of the five highest scoring AlphaFold models of the TASOR SPOC domain (blue) in complex RNA Pol II CTD di-heptapeptides phosphorylated at Ser2 (upper panel) or Ser5 (lower panel).

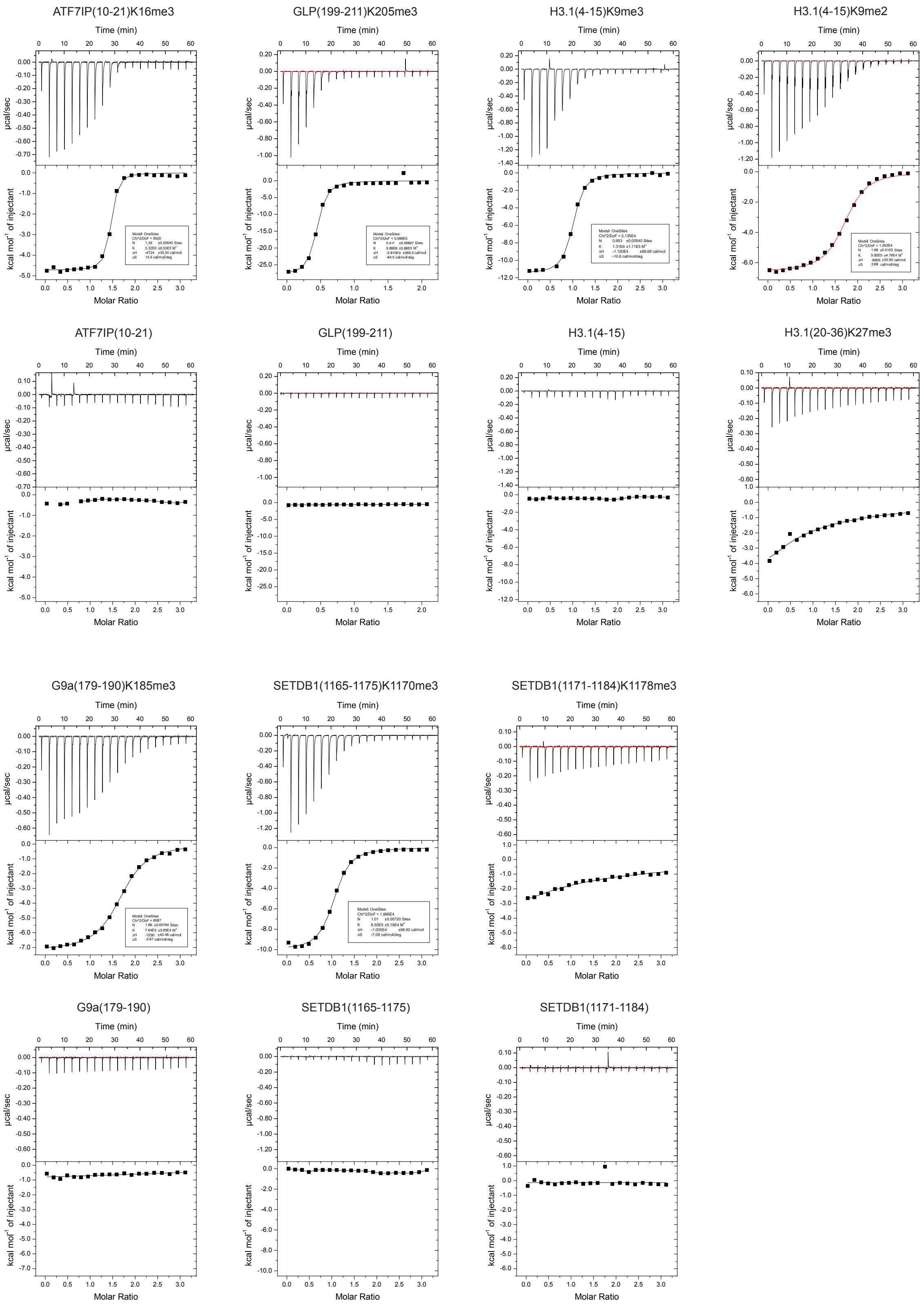

Fig. S4. ITC isotherms and thermograms for titration of H3K9 and H3K9-like peptides, lysine-methylated or unmethylated, into a solution of the MPP8 chromodomain (CD).

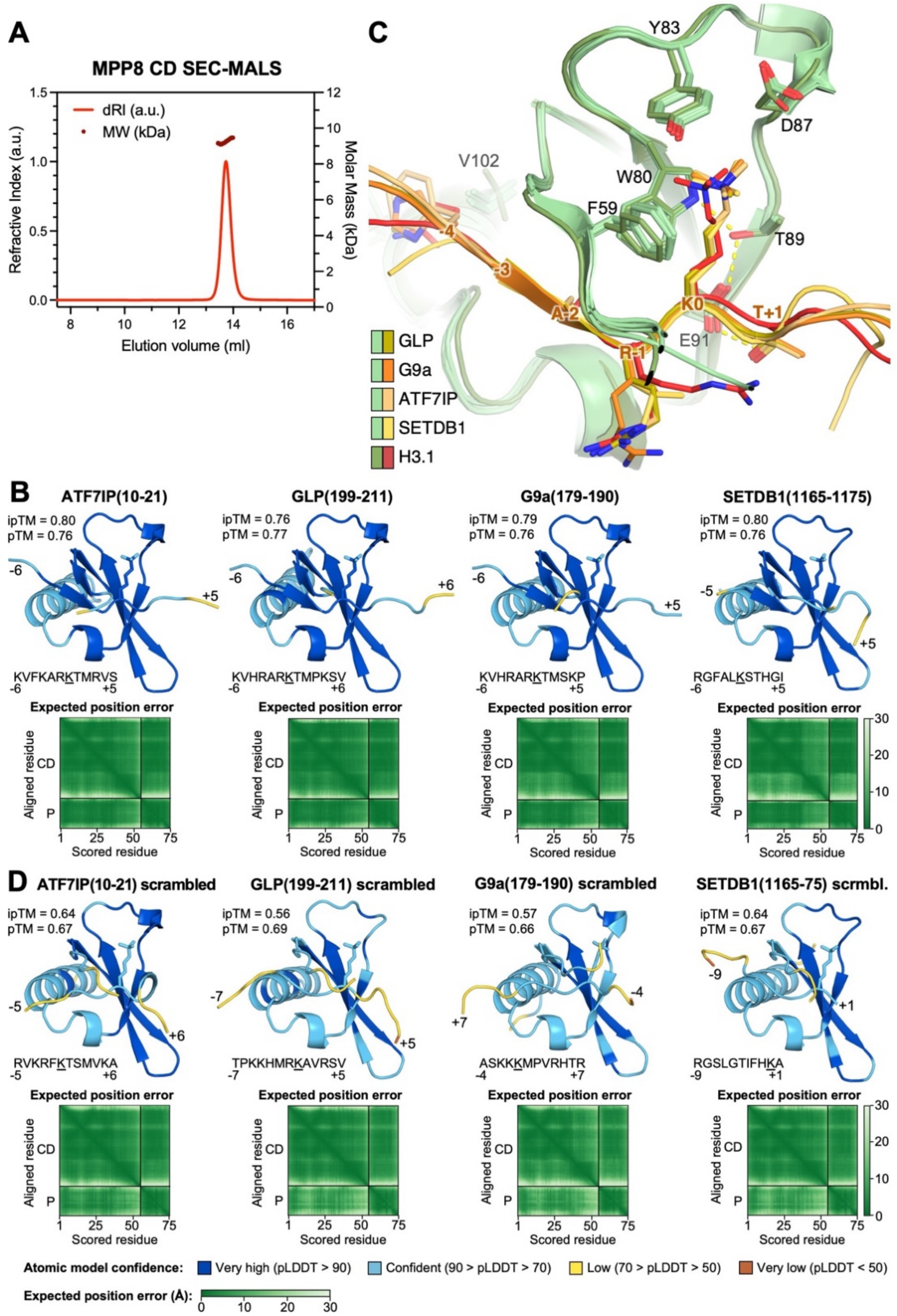

Fig. S5. Oligomerization state of MPP8 CD and structural modeling in complex with H3K9-like peptides with AlphaFold3. (**A**) Size-exclusion chromatography with multiangle light scattering (SEC-MALS) of MPP8 CD. dRI, relative refractive index. MW, molar mass. (**B**) AlphaFold3 predictions with trimethylated H3K9-like peptides. Upper panels: atomic models of the chromodomain-peptide (CD-P) complexes colored by model confidence (pLDDT). Lower panels: expected position error plots. (**C**) Closeup of the interactions between MPP8 CD and H3K9me3 or H3K9-like peptides. (**D**) Atomic models and expected position error plots from AlphaFold3 runs using a scrambled (randomized) amino acid sequence of each peptide in (B). The terminal residues of each peptide are numbered by distance from the trimethyl-lysine residue (underlined in each peptide sequence).
